# Cystatin C takes part in melanoma-microglia cross-talk: possible implications for brain metastasis

**DOI:** 10.1007/s10585-018-9891-0

**Published:** 2018-05-02

**Authors:** Adi Moshe, Sivan Izraely, Orit Sagi-Assif, Roshini Prakash, Alona Telerman, Tsipi Meshel, Thomas Carmichael, Isaac P. Witz

**Affiliations:** 1 Department of Cell Research and Immunology, George S. Wise Faculty of Life Sciences, Tel Aviv University, 69978 Tel Aviv, Israel; 2 Department of Neurology, David Geffen School of Medicine, UCLA, Los Angeles, CA USA

**Keywords:** Brain metastasis, Cystatin C, Melanoma, Microglia, Stroke

## Abstract

The development of melanoma brain metastasis is largely dependent on mutual interactions between the melanoma cells and cells in the brain microenvironment. Here, we report that the extracellular cysteine protease inhibitor cystatin C (CysC) is involved in these interactions. Microglia-derived factors upregulated CysC secretion by melanoma. Similarly, melanoma-derived factors upregulated CysC secretion by microglia. Whereas CysC enhanced melanoma cell migration through a layer of brain endothelial cells, it inhibited the migration of microglia cells toward melanoma cells. CysC was also found to promote the formation of melanoma three-dimensional structures in matrigel. IHC analysis revealed increased expression levels of CysC in the brain of immune-deficient mice bearing xenografted human melanoma brain metastasis compared to the brain of control mice. Based on these in vitro and in vivo experiments we hypothesize that CysC promotes melanoma brain metastasis. Increased expression levels of CysC were detected in the regenerating brain of mice after stroke. Post-stroke brain with melanoma brain metastasis showed an even stronger expression of CysC. The in vitro induction of stroke-like conditions in brain microenvironmental cells increased the levels of CysC in the secretome of microglia cells, but not in the secretome of brain endothelial cells. The similarities between melanoma brain metastasis and stroke with respect to CysC expression by and secretion from microglia cells suggest that CysC may be involved in shared pathways between brain metastasis and post-stroke regeneration. This manifests the tendency of tumor cells to highjack physiological molecular pathways in their progression.

## Introduction

Brain metastasis is common in patients with malignant melanoma [1–4]. The progression of melanoma towards brain metastasis is determined by various factors [5, 6] including reciprocal interactions between melanoma cells and cells in the brain microenvironment and their soluble products [7]. For example, the interaction between brain-metastasizing melanoma cells and astrocytes promoted the formation of a pro-inflammatory milieu in the brain, thereby promoting the formation of metastasis in this organ [8].

Ongoing studies on interactions between brain metastasizing melanoma cell and microglia (unpublished) are focused on the functional reprogramming of the microglia by melanoma cells and their soluble products. Preliminary experiments suggested that the melanoma-microglia cross-talk stimulated an enhanced secretion of the cysteine protease inhibitor Cystatin C (CysC) (unpublished). This protein also functions as an antagonist of TGFβ by interacting with the TGFβ type II receptor [9].

CysC was previously indicated to be involved in the formation of several tumor types. In vivo studies showed that over-expression of CysC led to a reduction of fibrosarcoma lung metastasis in mice [10]. CysC over-expressing B16-F10 melanoma cells reduced lung colonization, increased apoptosis of micrometastases and increased survival in mice [11]. In contrast, other studies suggested that CysC exerts pro-malignancy functions: a correlation was found between increased serum CysC and metastatic melanoma and colorectal cancer [12]. CysC deficient mice (homozygous null-allele) were tested for the formation of lung metastases induced by B16-F10 cells. The knockout mice had significantly fewer lung metastases than wild type mice [13].

Here, we investigated the role of CysC in the progression of brain-metastasizing melanoma cells.

## Materials and methods

### Cell cultures

The production and maintenance of melanoma brain metastasizing variant YDFR.CB3 and cutaneous human melanoma variant YDFR.C were described previously [14]. The production and maintenance of melanoma cutaneous human melanoma variant DP.C were previously described [15]. Metastasizing variants used in this study (DP.CB3, DP.CB4) were produced as described previously for other DP model variants [15].

Immortalized human brain microvascular endothelial cell line (BEC) were kindly provided by Dr. Clara Nahmias and Prof. Pierre-Olivier Couraud (Inserm, U1016, Institut Cochin, Paris, France) and were maintained as previously described [14].

Immortalized human microglia-SV40 cell line was purchased from ABM (ABM, Milton, Canada) and were maintained as previously described [15].

### Construction of mCherry/EGFP-expressing cells

To produce mCherry- expressing melanoma cells (YDFR.C, YDFR.CB3, DP.CB2 and DP.CB4), cells were transfected with a pQCXIP-mCherry plasmid. To produce EGFP—expressing YDFR.CB3, cells were transfected with a pQCXIP-EGFP plasmid. Transfected cells were selected using 1 µg/ml puromycin (InvivoGen, San Diego, CA, USA). The transfection procedure was previously described [15].

### Downregulation of CysC expression

The downregulation of CysC was constructed using pGIPZ vectors (Thermo Fisher Scientific) containing shRNA sequences targeting human CST3 mRNA (NM_000099.3). For the preparation of melanoma-shCysC cells, a combination of four vectors was used (V3LHS_321136, V3LHS_321137, V3LHS_321139, V3LHS_321140) to transfect YDFR.CB3 cells. For the preparation of MG-shCysC cells, V3LHS_3211339 was used to transfect microglia. The cells were produced as previously described [16]. A sh-non-silencing pGIPZ vector (RHS4531) was used as a negative control (shControl). Transfected cells were selected using 1 µg/ml (YDFR-CB3) or 6 µg/ml (MG) puromycin (InvivoGen, San Diego, CA, USA).

### Preparation of conditioned medium

5 × 10^5^ of melanoma or cells (melanoma, microglia or BEC) were cultured for 24 h. The cells were washed with PBS and grown in starvation medium for 24 h. Conditioned medium (CM) was collected, centrifuged for 5 min at 1400 rpm and filtered (0.45 µm, Whatman GmbH, Dassel, Germany). 0.5% FCS supplemented medium was used for starvation in all the experiments.

### Immuno-detection of secreted CysC

For experiments of CM treatments, 5 × 10^5^ of cells (melanoma, microglia or BEC) were cultured for 24 h. The cells were washed with PBS and incubated for 4 h with CM or with the starvation medium as control. The cells were then grown for 20 h in a suitable starvation medium.

For co-culture experiments, microglia and melanoma cells were seeded. The two cell types were seeded either separately (5 × 10^5^ cells per plate) or together (co-culture, 2.5 × 10^5^ of each cell type per plate). Cells were washed with PBS and cultured for 24 h in starvation medium (50% PriGrow III, 50% RPMI).

For experiments of hypoxia treatments, 5 × 10^5^ of cells (microglia or BEC) were cultured for 24 h. The cells were washed with PBS and incubated with DMEM starvation medium lacking glucose (hypoxia) for 4 h in a hypoxia chamber with a gas mixture of 1% O_2_, 5% CO_2_ and 94% N_2_. As control, cells were incubated for 4 h with DMEM starvation medium under normal glucose and O_2_ conditions (normoxia). The cells were then grown for 20 h in a suitable starvation medium.

Secreted protein analysis was done for all experiments as follows: cell supernatants preparation was performed by centrifugation for 5 min at 1400 rpm and filtration (0.45 µm, Whatman GmbH, Dassel, Germany). 20 µl of the supernatant were used for western blot analysis as according to standard procedure, using anti human CysC polyclonal antibody (R&D systems, Minneapolis, MN, USA). Each experiment was repeated 3–5 times.

### Scratch wound healing assay

Melanoma cells were seeded on a collagen-coated 96-well plate (YDFR.CB3: 4.5 × 10^4^ cells per well. YDFR.C: 5 × 10^4^ cells per well). On confluence, the cell monolayer was scratched using a 96-well WoundMaker. Cells were washed twice with RPMI medium. Starvation medium (0.5% RPMI) was added to the cells with 20 nM recombinant human CysC (R&D systems, Minneapolis, MN, USA). Starvation medium was used as control. The plates were imaged every 2 h for 72 h, and images were analyzed using the IncuCyte system (Essen BioScience, Ann Arbor, MI, USA). Each experiment was repeated 3 times.

### Trans-endothelial migration through a BBB model

Trans-endothelial migration assays were done as previously described with minor modifications [15].

For mCherry-melanoma migration assays, 1 × 10^5^ cells were loaded onto endothelial monolayer-seeded transwells (8 µm; Corning Costar Corp.) with or without CysC inactivating antibody. Cells were allowed to migrate for 24 h.

For CysC-silenced melanoma migration assays, 5 × 10^4^ mCherry-expressing YDFR.CB3 and 5 × 10^4^ melanoma-shCysC or melanoma-shControl were loaded onto the endothelial monolayer and allowed to migrate for 24 h.

For migration assay of mCherry-expressing YDFR.CB3 towards microglia, MG-shCysC or MG-shControl were seeded on 24-well plate and were grown in starvation medium for 48 h prior to the addition of the transwells seeded with BEC and melanoma cells.

Each experiment consisted of 3 repeats for each treatment. The experiments were repeated 3–4 times. Fixation, imaging of transwells and statistical analysis was done as previously described [15].

### Migration of microglia through extracellular matrix

1 × 10^5^ microglia cells were loaded onto collagen-coated transwell inserts (8 µm; Corning Costar Corp., New York, NY, USA) and allowed to migrate for 24 h. MG-shControl or MG-shCysC cells were allowed to migrate towards MCM or YDFR.CB3. Melanoma cells were seeded (1 × 10^5^ cells in 24-well plate) and starved for 24 h previous to the addition of microglia-loaded transwells.

Microglia cells were allowed to migrate towards melanoma-shControl or melanoma-shCysC cells. Melanoma cells were seeded (1 × 10^5^ cells in 24-well plate) and starved for 48 h previous to the addition of microglia-loaded transwells.

Each experiment consisted of 3 repeats for each treatment. The experiments were repeated 3–4 times. Fixation, imaging and calculation of migrated cells was done as previously described [15].

### Three dimensional cell culture (3D)

Melanoma cells cultured in Millicell inserts (Merck KGaA, Darmstadt, Germany) on 6-well plate. Firstly insert PET transparent membranes were pre-coated with matrigel/10% RMPI medium (1:1) and incubated at 37 °C for 30 min. Melanoma cells were resuspended in medium containing matrigel (1:4) and transferred to the coated insert. The insert was then placed on a 6-well plate containing 0.5% RMPI. Images were taken under a light microscope.

### In vivo model of stroke and brain metastasis

In vivo animal surgeries were performed in accordance with the NIH animal protection guidelines and protocols approved by University of California, Los Angeles Chancellor’s Animal Research Committee. NOD-scid gamma (NSG), a highly immunodeficient mice lacking T, B and NK cells, was used to mimic stroke-like experiments in vivo (Jackson Laboratories). Inducing strokes in this immunodeficient mouse strain provides a mouse model that allows for the engraftment of human cancer cells without graft rejection and little to no involvement of innate or acquired immunity [17, 18].

Adult male NSG mice aged 2–4 months were subjected to ischemic strokes. Briefly, animals were anesthetized with isoflurane (2.5–3%) and positioned on a stereotactic frame (Model 940, David Kopf Instruments). A midline cut on the head was performed followed by a transverse cut above the zygomatic arch. A craniotomy exposed the left middle cerebral artery. The proximal branch of the middle cerebral artery was cauterized followed by a 15 min bilateral occlusion of the jugular veins. Mice body temperature was maintained at 37 ± 0.5 °C during the course of the surgery determined using a rectal probe (RightTemp™, Kent Scientific, Torrington, CT). After cauterization, cut portions of the skin and the muscles were was glued together to cover the skull and animals were returned to their respective cages for recovery. Metastasis was induced 7 days after stroke by injecting EGFP- expressing YDFR-CB3 cells (10^6^ cells/ 50 µl of L15 medium) intracardially (a period chosen based on peak regeneration/tissue repair occurring after stroke) [19, 20]. Fourteen days after stroke animals were sacrificed and the brains were processed for immunostaining.

### Immunohistochemistry of mice brain sections

Immunohistochemistry was performed on 50 µm brain sections. Extracted brains were fixed in 4% paraformaldehyde for 24 h and then permeabilized until they sank in 30% sucrose. The brains were then cryosectioned to 50 µm thick sagittal sections and washed with PBS and blocked with 2% horse serum and 5% normal donkey serum in 0.3% Triton. Primary antibodies included Iba-1 anti-goat and CysC anti-rabbit (Abcam, Cambridge, MA), PECAM anti-rat (BD Biosciences, San Jose, CA). Sections were incubated with primary antibodies overnight followed by wash with PBS. Sections were stained with respective secondary antibodies, incubated for 1 h, and washed 3 times with PBS. The stained sections were mounted on slides, dehydrated and coverslipped. The sections were then imaged at 100× on Nikon confocal microscope.

### Statistical analysis

Data were analyzed using Student’s *t* test and considered significant at p values ≤ 0.05. Bar graphs represent mean and standard deviation (SD) across multiple independent experimental repeats.

## Results

### Melanoma and microglia reciprocally stimulate CysC secretion

**Fig. 1:**
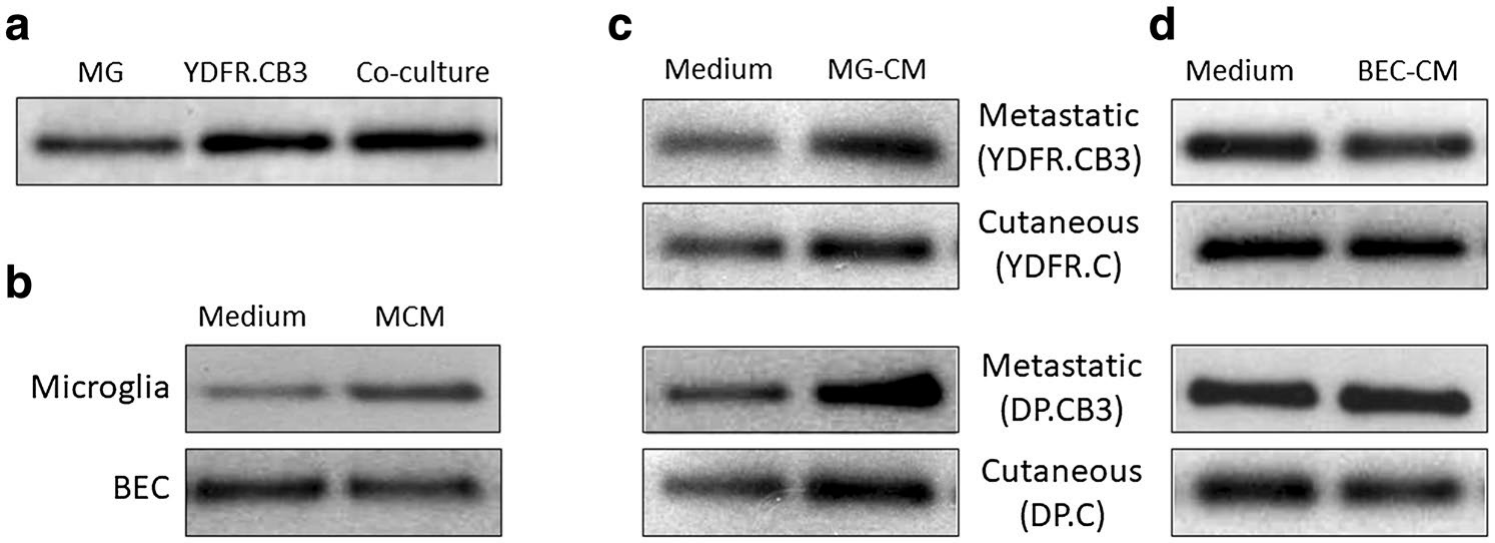
CysC secretion patterns from microglia, BECs and melanoma cells. **a** Microglia cells (5 × 10^5^), metastatic melanoma cells (5 × 10^5^), and a co-culture of microglia (2.5 × 10^5^) and metastatic melanoma cells (2.5 × 10^5^) were cultured for 24 h. **b** Microglia cells and BEC were treated with MCM. Treatment with starvation medium was used as control (Medium). **c** and **d** Melanoma cells were treated with MG-CM (**c**) or with BEC-CM (**d**). Treatment with starvation medium was used as control (Medium). Melanoma cells tested: metastatic (YDFR.CB3, DP.CB3) and cutaneous (YDFR.C, DP.C). Western blot was applied to detect CysC (14 kD) in the cell culture supernatants

Ongoing studies are aimed to identify molecular changes occurring in brain microenvironmental cells that are induced by brain-metastasizing melanoma cells. It was found that the secretome of melanoma-microglia co-cultures contained higher levels of the extracellular cysteine protease inhibitor CysC than the secretome of each cell when cultured separately (Fig. 1a). This result suggested that at least one of the cell types secreted more CysC as a result of cell to cell contact with the other cell type.

In order to determine which of the cell types secreted more CysC following contact with the other cell type, we treated each cell type separately with conditioned medium (CM) of the other cellular partner. Melanoma CM (MCM) was prepared from YDFR.CB3 cells which is a metastatic variant of the human YDFR melanoma cell line [21]. Western blot analysis (Fig. 1b) indicated that treatment of microglia cells with MCM led to an increased secretion of CysC from these cells compared with control cells treated with fresh medium. The reciprocal experiment, melanoma cells treated with microglia CM (MG-CM), showed that melanoma cells treated with MG-CM secreted more CysC than control melanoma treated with fresh medium. Similar results were obtained when both metastatic and cutaneous melanoma variants from two different human melanoma cell lines (YDFR and DP) were used (Fig. 1c). Our results show that microglia and melanoma cells upregulate each other’s CysC secretion.

Since the interaction of metastasizing melanoma cells with the blood–brain barrier is a pivotal step in metastasis formation in the brain, we asked whether melanoma cells are capable of altering CysC secretion from brain microvascular endothelial cells (BEC). In contrast to microglia cells, MCM treatment did not lead to an increase in CysC secretion from the BEC (Fig. 1b). Reciprocal experiments testing the effect of CM of BEC on CysC secretion from melanoma cells yielded similar results: BEC had no effect on CysC secretion from melanoma cells. This was confirmed for both the metastatic and the cutaneous melanoma cell variants (Fig. 1d).

### CysC shapes the malignancy phenotype of melanoma cells

The aim of the next set of experiments was to establish whether CysC exerts functions that contribute to the malignancy phenotype of melanoma cells.

CysC secreted spontaneously from both melanoma cells as well as from interacting microenvironmental cells masks effects of exogenously added CysC (unpublished). We employed therefore in some of the experiments described in this section, neutralizing anti CysC antibodies which, by neutralizing endogenously secreted CysC, enable to evaluate the functional effects of extracellular CysC.

#### CysC enhanced the migratory capacity of brain-metastasizing melanoma cells (wound healing assays)

**Fig. 2:**
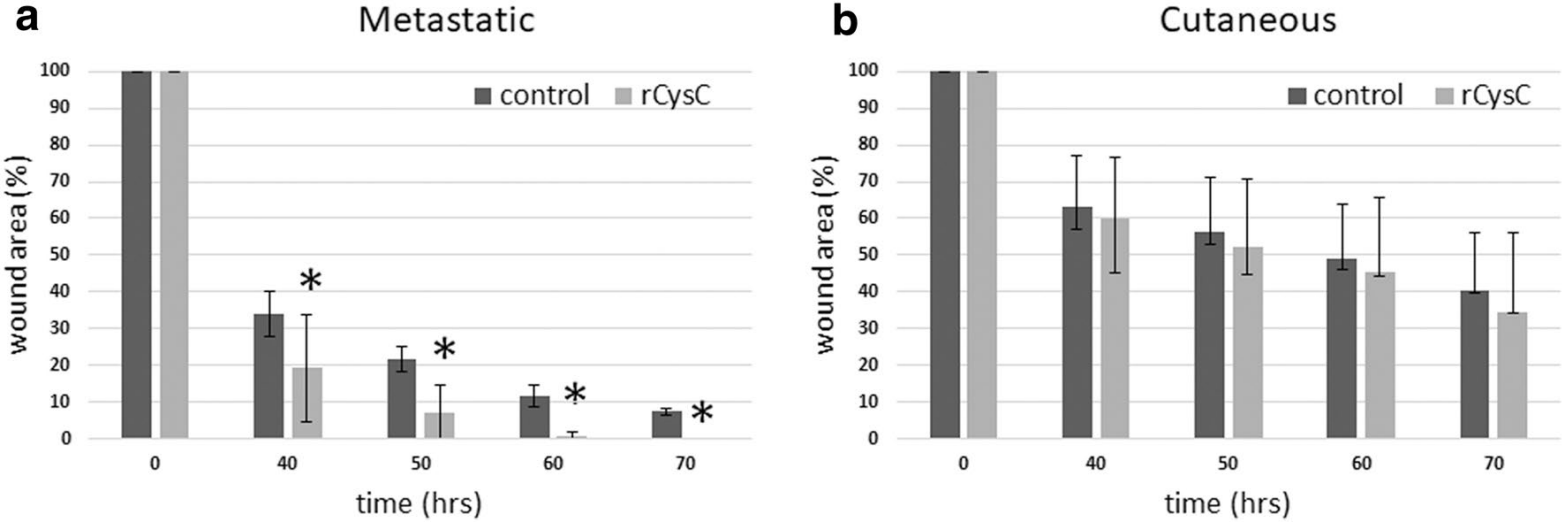
CysC enhances in vitro wound healing of metastatic melanoma. Metastatic (**a**) and cutaneous (**b**) melanoma monolayers were scratched and treated with recombinant CysC (rCysC). Control cells were grown in starvation medium. Graphs show the relative size of wound area (%) as a function of time (h). Bars represent the average % wound area ± SD of three independent experiments. *p < 0.05

Tumor cell migration is a crucial step in metastasis formation. Since secreted proteins in the tumor microenvironment may affect tumor cell migration, we employed the in vitro wound-healing assay to measure CysC effects, if any, on melanoma cell migration. We first determined that recombinant CysC (rCysC) did not affect melanoma cell viability (data not shown). It was then demonstrated that rCysC enhanced the wound-healing capacity of metastatic YDFR.CB3 cells compared to untreated controls. The difference between treated and control cells started to show 10 h post wounding and became more prominent with time. The wound of rCysC-treated melanoma cells closed completely at 60 h post wounding, while at this time point only 90% of the wound of control cells was closed (Fig. 2a).

In vitro wound healing assays were also performed with the cutaneous variant of the YDFR melanoma. No significant differences were found in wound healing capacity rate for these cells treated with recombinant CysC (Fig. 2b).

#### CysC enhances melanoma trans-endothelial migration

The trans-migration of tumor cells through the blood–brain barrier (BBB) is a crucial step in the establishment of brain metastasis [6, 22]. Since our results indicated that CysC enhances the migration of melanoma cells, we tested its effect on the transmigration of mCherry-expressing melanoma through a layer of BEC. We used an experimental model of the BBB consisting of cell-permeable transwells seeded with human BEC. Four mCherry-expressing melanoma variants were used: a cutaneous variant (YDFR.C), the metastatic variant of the same melanoma (YDFR.CB3) and two metastatic variants of the DP melanoma (DP.CB2 and DP.CB4).

The untreated YDFR.C cutaneous variant migrated less well than the untreated three metastatic variants. Exposure of the four melanoma cell variants to rCysC did not affect the trans-migration of these cells (not shown). This lack of CysC effect could be explained by the fact that both melanoma cells, as well as BEC, spontaneously secrete CysC (Fig. 1). Saturating levels of spontaneously secreted CysC may mask the effect of the exogenous recombinant protein on the migratory capacity of the cells.

**Fig. 3:**
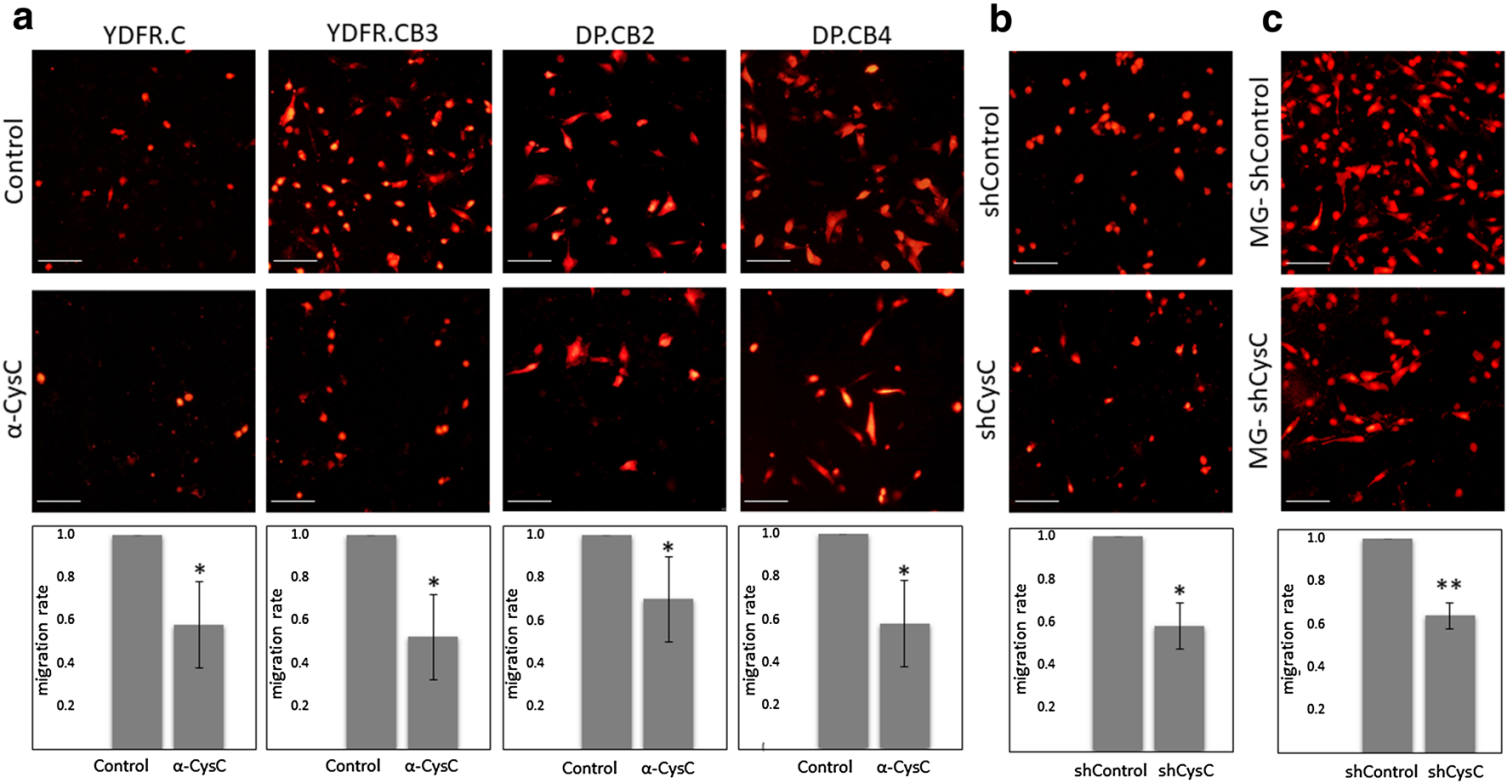
CysC enhances melanoma migration through a brain endothelial barrier. **a** mCherry-expressing melanoma variants migrated through BEC in the absence (control) or presence or of anti-CysC neutralizing antibody (α-CysC). Representative images for each treatment are shown. Red: melanoma cells. **b** Trans-endothelial migration of mCherry expressing melanoma cells mixed with melanoma-shControl cells (shControl) or with melanoma-shCysC cells (shCysC). **c** Trans-endothelial migration of mCherry-expressing melanoma cells (YDFR.CB3) towards MG-shControl cells or MG-shCysC cells. Representative images for each treatment are shown. Red: mCherry-expressing melanoma cells. Bar: 100 µm. Graphs represent the average and standard deviation of the relative migration of mCherry-expressing cells ± SD of three independent experiments. The relative migration in each experiment was calculated compared to control. *p < 0.05, **p < 0.01

In view of the above we studied the effect of exogenous CysC on trans-endothelial migration of melanoma cells by inhibiting its spontaneous secretion. Neutralization of spontaneously-secreted CysC by neutralizing antibodies led to a significant decreased transmigration of all four melanoma variants across the BEC layer, demonstrating that CysC enhances trans-endothelial migration (Fig. 3a).

To further validate the finding that CysC enhances the transmigration of melanoma cells through the BBB, we silenced CysC expression in brain-metastasizing YDFR.CB3 cells by CysC specific shRNA to yield melanoma-shCysC cells. Control (melanoma-shControl) cells were infected with a non-silencing shRNA. A 1:1 mixture of mCherry expressing YDFR.CB3 cells and melanoma-shCysC cells secreted less CysC than a 1:1 mixture of mCherry expressing YDFR.CB3 cells and melanoma-shControl cells (not shown). The transendothelial migration of the mixture of mCherry-expressing melanoma cells with melanoma-shCysC cells across a BEC layer was reduced compared to the migration of the mCherry-expressing melanoma cells/ melanoma-shControl mixture (Fig. 3b). These results supported the above conclusion that CysC promotes migration of melanoma cells across the BBB.

Next, we tested whether similarly to melanoma-derived CysC, microglia-derived CysC will also promote melanoma transmigration through the BBB. CysC-silenced microglia cells (MG-shCysC) that secrete less CysC than control MG-shControl cells were used in these experiments. The trans-endothelial migration of mCherry expressing YDFR.CB3 melanoma cells towards MG-shCysC cells was lower than towards MG-shControl (Fig. 3c). These results indicated that the CysC secreted from microglia cells and that secreted from melanoma cells function similarly in promoting transendothelial migration of the tumor cells.

Taken together the above data indicate that CysC is a positive regulator of melanoma cell invasion through the brain endothelial barrier.

### CysC inhibits the migration of microglia cells towards melanoma cells

**Fig. 4:**
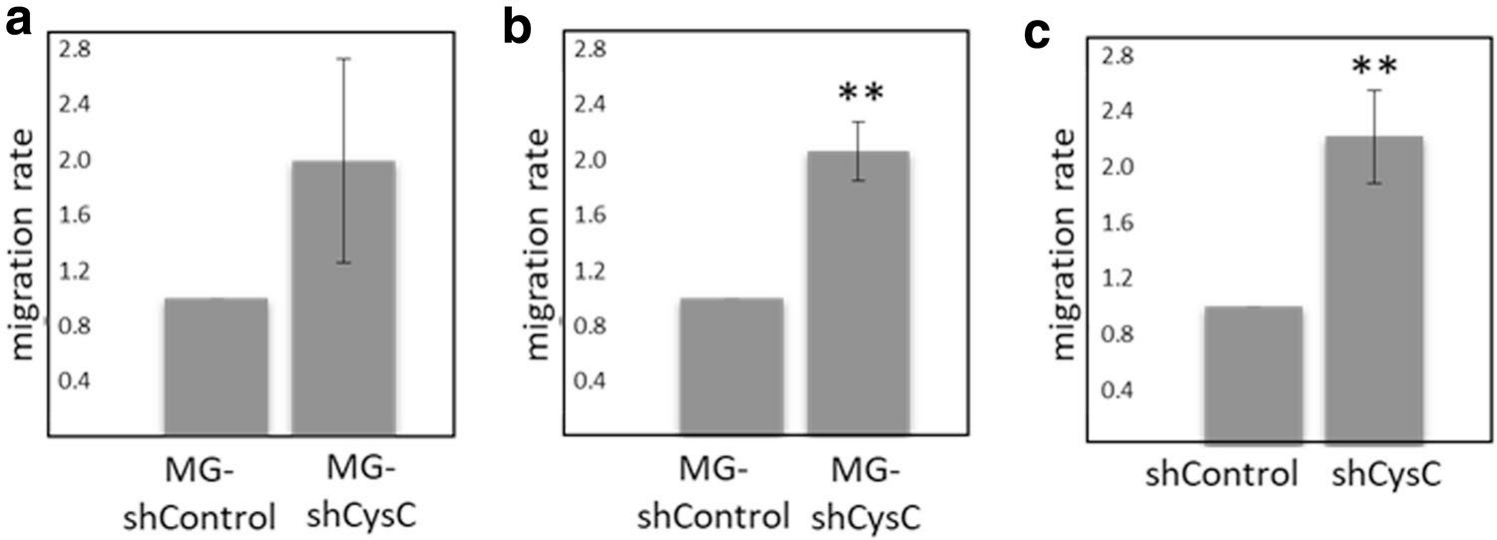
CysC inhibits microglia migration towards melanoma. **a** and **b** Migration of MG-shControl or MG-shCysC cells towards MCM (**a**) or melanoma (YDFR.CB3) cells (**b**). **c** Migration of microglia towards melanoma-shControl cells (shControl) or melanoma-shCysC cells (shCysC). Representative images for each treatment are shown. Blue: microglia cells. Bar: 100 µm. Graphs represent the average and standard deviation of the relative migration of microglia cells ± SD of three independent experiments. The relative migration in each experiment was calculated compared to control. **p < 0.01

These experiments were aimed to establish whether CysC affects microglia cell migration. In these migration assays, we used collagen-coated transwells. MG-shCysC or MG-shControl cells were allowed to migrate towards MCM for 24 h. MG-shCysC cells migrated better than control cells, suggesting that CysC exhibits an inhibitory effect on microglia cell migration towards melanoma-derived soluble factors (Fig. 4a). The migration of microglia towards melanoma cells was then tested. As in the previous experiment, MG-shCysC cells migrated better than MG-shControl cells towards metastatic melanoma cells, providing further proof that CysC inhibits migration of microglia cells (Fig. 4b).

Next, we tested the migration of microglia cells towards melanoma-shCysC cells or melanoma-shControl cells. The results demonstrated that microglia cells migrated better towards melanoma-shCysC cells than towards melanoma-shControl cells (Fig. 4c), providing further proof for the inhibitory function of CysC on microglia migration.

### CysC accelerates the formation of melanoma spheroids

Previous unpublished work in our lab showed that the in vivo malignant phenotype of melanoma cells correlates with their ability to form three-dimensional (3D) spheroids in vitro. More highly malignant cells formed spheroids faster than less malignant ones.

**Fig. 5:**
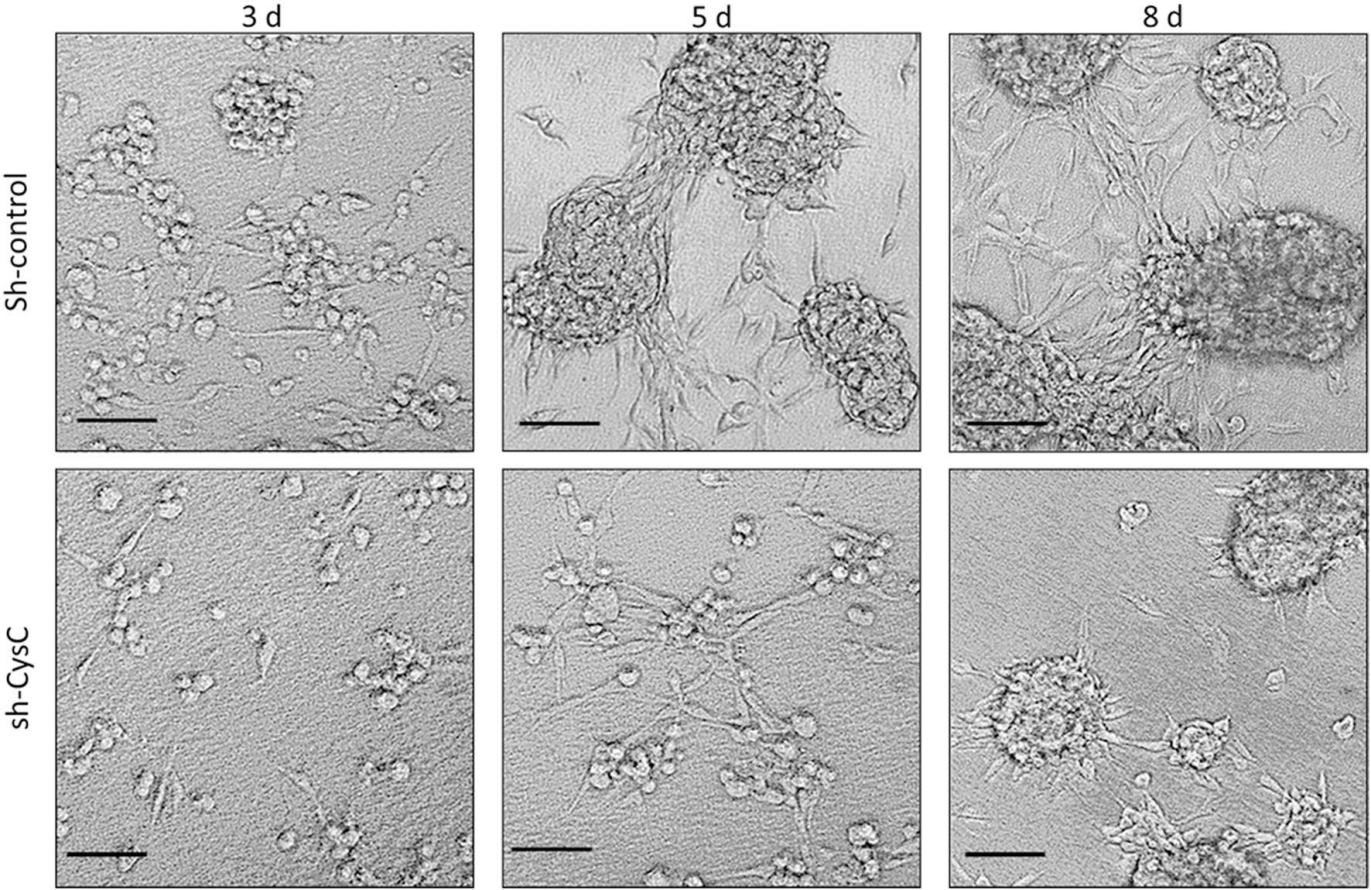
CysC enhances the formation of melanoma-three dimensional spheroids. Melanoma-shControl cells (shControl) or melanoma-shCysC cells (shCysC) were grown on three dimensional matrigel for 8 days. Representative images for 3, 5 and 8 days are shown. Bar: 100 µm

Melanoma-shCysC or melanoma-shControl cells were used in these experiments to determine if CysC is involved in the formation of 3D structures. These cells were seeded on 3D matrigel [23]. The cells were then inspected for morphological alterations at different time points (Fig. 5). 24 h following seeding, both melanoma-shControl as well as melanoma-shCysC cells still appeared as isolated cells. Three days post seeding, the melanoma-shControl cells appeared as large clusters and tube-like structures were seen to emerge. At this time point, melanoma-shCysC cells started to form small clusters. At 5 days after seeding, melanoma-shControl cells formed very large spheroids, connected by tube-like structures. Melanoma-shCysC cells were still found in small clusters, accompanied by some tube -like structures. At 8 days after seeding, the large 3D spheroids formed by melanoma-shControl cells were connected by complex tube-like structures. Melanoma-shCysC cells on the other hand formed intermediate spheroids, connected by thin tube-like structures.

These results demonstrate that CysC accelerates the in vitro formation of melanoma 3D spheroids and as such may promote melanoma malignancy, since 3D multicellular tumor spheroid systems are used to model in vivo tumor growth [24].

### Stroke upregulates CysC in the brain of post-stroke mice and in the secretome of microglia cells

In view of the findings that CysC regulates some phenotypic traits of microglia as well as those of brain-metastasizing melanoma cells, it was of interest to determine if this cytokine is involved in pathological processes occurring in the brain. Preliminary experiments involving post-stroke neuro-regeneration and brain metastasis suggest that CysC is involved in these processes.

**Fig. 6:**
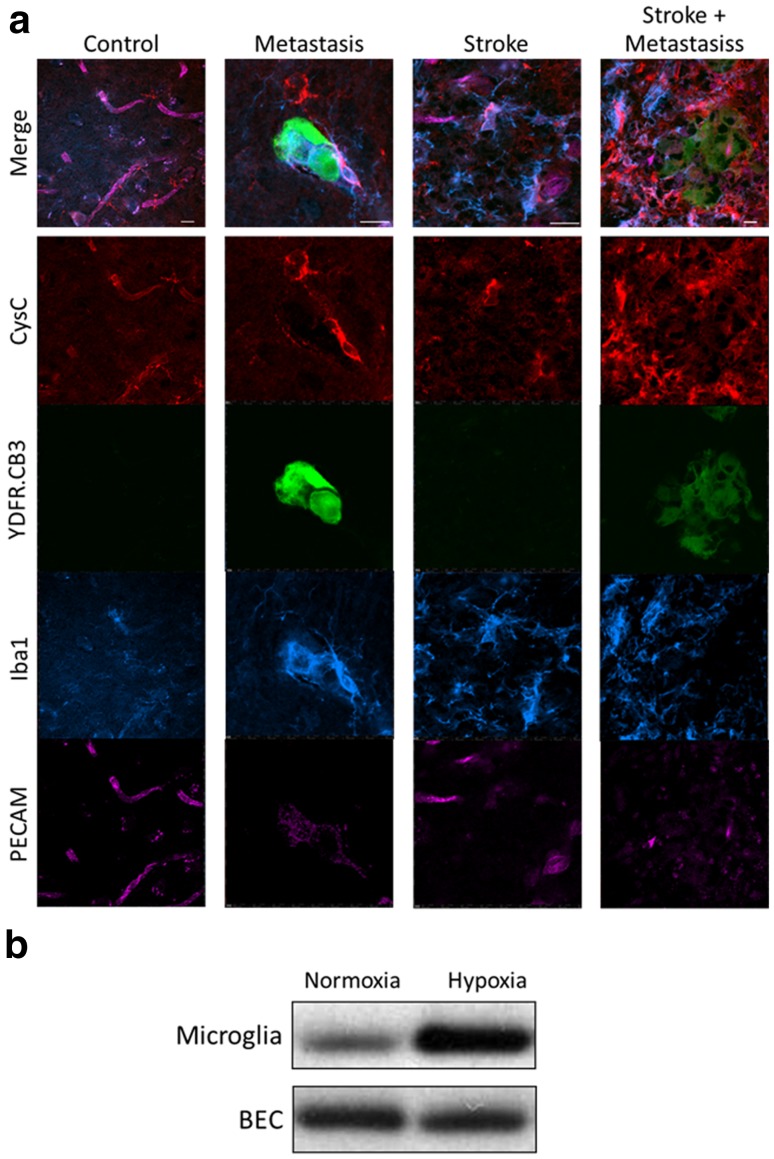
Increased CysC expression in the brain of mice bearing xenografted melanoma brain metastasis or in post-stroke regenerating brain. A. IHC of brain sections. Red: CysC. Green: YDFR.CB3 cells. Blue: Iba1 (microglia marker). Pink: PECAM (endothelial cell marker). Bar: 10 µm. B: Microglia cells and BEC were subjected to oxygen and glucose deprivation (hypoxia) or normal oxygen and glucose (normoxia). Western blot was applied to detect CysC in the secretome of the brain cells

Immunostaining of mouse brain sections showed a high vasculature expression of CysC, as reported [25, 26]. In the brain of mice bearing melanoma metastasis, as well as in the brain of post-stroked mice, the vasculature expression of CysC was reduced, while the expression around microglia-rich areas was increased. The increase in CysC expression around the microglia was even more pronounced in the brain of post-stroke mice that were also bearing melanoma brain metastasis (Fig. 6a).

In order to find out which brain cells are responsible for the increase in CysC levels in the brain of post-stroke mice, we used an in vitro “stroke” model system. Cells were subjected to oxygen and glucose deprivation (hypoxia) or to normal oxygen and glucose as control (normoxia). Microglia cells subjected to hypoxia, secreted more CysC than control cells subjected to normoxia. (Fig. 6b). The exposure of BEC to hypoxia did not lead to any change in CysC secretion (Fig. 6b). These results suggest that the increased levels of CysC in the brain of post stroke mice may be, at least partially, microglia-derived.

## Discussion

Cells and molecules in the tumor microenvironment may exert yin yang functions either supporting or inhibiting tumor progression [27–29]. CysC and the microglia cells producing it may belong to this group of microenvironmental components. Based on the results of this study we propose the following working model with respect to the involvement of CysC in melanoma brain metastasis: the encounter between melanoma cells and microglia reciprocally upregulates the secretion of CysC from both types of cells. This in turn, reprograms both types of the interacting cells: the migration and the inter-endothelial invasiveness of melanoma cells are augmented while the migratory capacity of the microglia cells is diminished.

Whereas the CysC-mediated enhancement of the migratory and invasive capacities of melanoma cells would promote their further spread, the CysC-mediated dampening of microglia migratory capacity could have opposing effects. If microglia cells exert pro-malignancy functions as had been reported in some cases [30], restricting their movement could be beneficial for individuals with brain metastasis. If, on the other hand, microglia cells exert protective functions against brain metastasis then restricting their movement would have a detrimental outcome.

In addition to the migration and invasion enhancing activities of CysC, this cytokine enhanced the formation of three-dimensional structures of melanoma cells, a parameter that, in our hands, serves as an in vitro measure of in vivo malignancy (unpublished).

CysC often exerts inhibitory effects on cell invasion and metastasis via its capacity to neutralize ECM-remodeling proteases [10, 11, 31]. Our results suggest that with respect to melanoma brain metastasis CysC may exert tumor-enhancing effects. This discrepancy can be explained by the multi-functionality of this cytokine. In addition to its protease inhibitory functions, CysC antagonizes TGFβ signaling in normal and cancer cells [9]. As such, CysC may modulate the TGFβ-mediated regulation of interactions between tumor cells and their microenvironment such as induction or repression of cytokines, growth factors and ECM proteins [32, 33] or the control of several tumor-promoting signaling pathways [34]. While the data of this study suggest that CysC positively regulates melanoma brain metastasis, further research is required to solve the mechanism by which CysC promotes melanoma brain metastasis.

The interaction between melanoma and microglia cells with respect to CysC secretion, seems to be unique to these interacting cells. The interaction of melanoma cells with BEC did not upregulate CysC secretion from either of these cells although both types of cells are capable of secreting CysC. The mechanism for this observation is under study.

The in vitro exposure of microglia to either melanoma-derived soluble factors or to oxygen and glucose deprivation enhanced the secretion of CysC from these cells, suggesting similarities in the response of cells in the brain microenvironment to signals delivered by stroke-inducing factors and by brain-metastasizing tumor cells. It is thus not unlikely that melanoma cells might utilize post-stroke repair mechanisms in order to establish brain metastasis. In vivo work supported these observations. Immunostaining of brain sections from stroked mice and of mice bearing melanoma brain metastasis indicated that both brain metastasis and stroke increased CysC expression in the brain.

## Abbreviations

3D: Three dimensional
CysC: Cystatin C
BEC: Brain microvascular endothelial cell
CM: Conditioned medium
MCM: Melanoma-conditioned medium
MG-CM: Microglia-conditioned medium
NSG: NOD-scid gamma mice
SD: Standard deviation
BBB: Blood–brain barrier
